# Prevalence of Echocardiographic Profile and Its Related Risk Factors in Congenital Heart Disease Patients; A Cross-Sectional Study

**DOI:** 10.31661/gmj.v13i.3517

**Published:** 2024-12-31

**Authors:** Ali Zolfi Ghol, Javad Rasouli, Shayan Atabaki Agdam, Mohammad Radvar

**Affiliations:** ^1^ Department of Pediatrics, School of Medicine, Urmia University of Medical Sciences, Urmia, Iran; ^2^ Department of Epidemiology and Biostatistics, School of Medicine, Urmia University of Medical Sciences, Urmia, Iran

**Keywords:** Congenital Heart Defects, Echocardiography, Epidemiology, Pediatrics, Patients

## Abstract

Background: Congenital heart disease (CHD) represents the most common and lethal birth defect affecting newborns. This study aimed to characterize the echocardiographic profile of CHDs, along with their prevalence and associated risk factors in CHD patients. Materials and Methods: This cross‐sectional, analytical study was conducted on CHD patients (aged less than 15) referred to the major pediatric hospital affiliated with Urmia University of Medical Sciences between March 2022 and October 2023. They were selected by convenience sampling method. All pediatric echocardiography was performed by a trained cardiologist. The collected data encompassed a comprehensive medical history, including mothers' parity, prior diagnoses of heart disease, hypertension, diabetes, thyroid disorders, COVID-19 infection during pregnancy, prior surgical procedures, use of complementary medicine during gestation, medication history, and mode of delivery. Additionally, child-related characteristics were investigated, including age, gender, co-existing congenital cardiovascular disease, neonatal intensive care unit (NICU) admission history, and echocardiographic findings. Specifically, the study focused on abnormalities in left ventricular fractional shortening (LVFS) and left ventricular ejection fraction (LVEF). Data analysis was done using SPSS software version 22.0 (IBM Corp., Armonk, NY, USA). Results: Of 293 CHD children, 59.72% were male and 40.27% were female. Overall, 132 (45%) children were below one year of age. Among echocardiography profile, Patent Foramen Ovale (PFO) constituted 65 cases accounting for 22.1% of all CHD cases and Atrial septal defect (ASD) was the second most common CHD accounting for 16.3% of all CHD cases. Mother's infection with Covid-19 during pregnancy (P0.001), type of delivery (P=0.015), and patient's NICU hospitalization (P=0.010) was statistically significant among the patients with normal and abnormal echocardiography. Conclusion: This study reveals a significant prevalence of Patent Foramen Ovale (PFO) and Atrial Septal Defect (ASD) among patients with congenital heart disease (CHD), highlighting the crucial role of early echocardiographic screening in this group. It also suggests a potential link between maternal COVID-19 infection during pregnancy, the method of delivery, and abnormal echocardiographic findings in CHD patients. These results underscore the need for further research into maternal and perinatal factors to better understand their impact on the development and prognosis of CHD, ultimately aiding in the improvement of clinical management and preventive strategies.

## Introduction

Congenital heart disease (CHD) encompasses a spectrum of structural malformations or functional abnormalities of the heart that are present at birth, although diagnosis may occur later in life [1]. The severity of these cardiac defects can range from undetectable anomalies to life-threatening conditions [2]. Global estimates suggest a CHD incidence of approximately 8 per 1,000 live births [1][3], with recent studies potentially revising this figure upwards to 9.5 per 1,000 [4]. Congenital heart disease (CHD) is the most common and severe type of congenital defect, significantly impacting the health and well-being of affected individuals. CHD encompasses various structural abnormalities of the heart, including defects such as patent foramen ovale (PFO) and atrial septal defect (ASD), which are among the most prevalent forms of this condition. In Iran, recent studies have reported a prevalence rate of approximately 7 to 12 per 1,000 live births, indicating a serious public health issue. This high prevalence underscores the necessity for comprehensive screening and early diagnosis to improve outcomes for affected children [4]. Despite advancements leading to a decrease in CHD-related mortality, it remains a significant contributor to infant and childhood mortality [5]. CHD can be categorized into two main types based on pathophysiology and the affected heart structures: acyanotic (ACHD) and cyanotic (CCHD) [6].

The etiology of most CHDs remains largely undetermined, likely involving a complex interplay between genetic susceptibility and environmental factors [7]. Established environmental risk factors include maternal infections during pregnancy, a family history of CHD, parity (number of pregnancies), maternal hypertension, and medication use during gestation [8]. Common clinical manifestations in neonates include tachypnea (rapid breathing), cyanosis (bluish skin discoloration), heart failure, and shock. Physical examination findings often include heart murmurs and absent femoral pulses [9]. Infants and children may present with dyspnea (breathlessness), digital clubbing, cyanosis, heart murmurs, syncope (fainting), episodes of squatting, heart failure, arrhythmias (irregular heart rhythms), and faltering growth [10]. CHD presents significant challenges for families, causing emotional distress, financial strain, and increased morbidity and mortality [11]. Early and accurate diagnosis is essential for improved outcomes and survival rates. Advancements in medical and surgical interventions have enabled a growing number of children with CHD to receive successful treatment and reach adulthood [12]. CHD represents a significant burden on both families and healthcare systems. Irrespective of the specific CHD type, these conditions can dramatically decrease the quality of life for all family members [13]. The financial strain is substantial due to the high healthcare costs associated with lifelong management. Individuals with CHD often require ongoing follow-up care and may undergo multiple surgical interventions throughout their lives [13]. Existing research within Iran suggests a CHD prevalence ranging from 4 to 8 per 1,000 live births [14]. This study addresses critical gaps in understanding the epidemiology and echocardiographic profiles of congenital heart disease (CHD) in the Iranian pediatric population. By focusing on prevalent forms such as Patent Foramen Ovale (PFO) and Atrial Septal Defect (ASD), and correlating them with maternal health factors, it provides valuable insights for clinical practice and public health policies. Additionally, the exploration of maternal COVID-19 infection's impact on CHD outcomes highlights the study's innovative approach, which may inform future research and improve early detection and treatment strategies for affected children in Iran. However, documented variations across the country highlight the need for more comprehensive epidemiological investigations, particularly within specific regions. To address this knowledge gap, the present study aims to analyze the echocardiographic characteristics profile of CHDs, their prevalence, and associated risk factors within the northwest region of Iran.

## Materials and Methods

Study Setting and Time

This study employed a cross-sectional, analytical design conducted between March 2022 and October 2023. The research setting was a major pediatric hospital affiliated with Urmia University of Medical Sciences. Prior to commencement, ethical approval was obtained from the relevant ethics review committee, ensuring adherence to the principles outlined in the Declaration of Helsinki. Informed consent was procured from the parents of all participating children. Data collection employed a convenience sampling method, targeting parents of children referred to the hospital's Heart clinic during the study period.

Ethical consideration

The study was conducted conscientiously, taking into account ethical considerations, and securing approval from the ethics committee at Urmia Medical Science University (Identification Code: IR.UMSU.REC.1400.167) while also obtaining written consent from all participants. Furthermore, the research adhered to the ethical guidelines for human experimentation outlined in the Helsinki Declaration.

Study Variables and Definitions

The study focused on several variables, including maternal clinical information, child-related characteristics, and echocardiographic findings. Maternal clinical information included parity (number of pregnancies), prior diagnoses of heart disease, hypertension, diabetes, thyroid disorders, COVID-19 infection during pregnancy, prior surgical procedures, use of complementary medicine during gestation, medication history, and mode of delivery. Child-related characteristics investigated were age, gender, co-existing congenital cardiovascular disease, neonatal intensive care unit (NICU) admission history, and echocardiographic findings. Specifically, the study assessed abnormalities in left ventricular fractional shortening (LVFS) and left ventricular ejection fraction (LVEF).

Study Arms and Clear-Cut Definition for a cross-sectional study

This study recruited inpatients within the pediatric hospital referred to Emergency ward for any diseases, encompassing neonates and children up to 15 years of age. All patients underwent echocardiograph. During data collection, we noted all pediatric echocardiography was performed by a trained cardiologist. Those patients with abnormal echocardiography suspected with congenital heart disease (CHD), which includes patients presenting with cyanosis (bluish skin discoloration), syndromic conditions (co-existing congenital anomalies), heart murmurs, faltering growth, respiratory distress, or arrhythmogenic cardiac disorders (irregular heart rhythms). The study excluded patients with critically unstable medical conditions, parents who declined informed consent.

Sample Size Consideration

Sample size was determined based on the convenience sampling method, targeting parents of children referred to the hospital's Heart clinic during the study period. A specific number of participants was not provided in the original text, so it would be beneficial to include that information if available.

Ethical Statement

Ethical approval was obtained from the relevant ethics review committee prior to the commencement of the study, ensuring adherence to the principles outlined in the Declaration of Helsinki. Informed consent was procured from the parents of all participating children, emphasizing the voluntary nature of participation and the right to withdraw at any time. Identification Code: IR.UMSU.REC.1400.167

Statistical Tests

Statistical analysis was performed using SPSS software version 22.0 (IBM Corp., Armonk, NY, USA). Categorical variables were measured as a percentage, while standard deviation and mean were used for quantitative variables. The Kolmogorov-Smirnov test was employed to assess the normal distribution of data. The Chi-square test (Fischer's exact test) and independent T-test (Mann-Whitney U) were utilized to compare categorical data between groups. P-values less than 0.05 were considered statistically significant.

## Results

**Table:** Table1. Demographic and clinical information of patients

Demographic Information	**N=293**	**%**
**Gender** Male Female	175 118	59.72 40.27
**Age** Below 1 year 1-3 3-6 6-12 12-15	132 26 34 72 29	45 8.9 11.6 24.6 9.9
**Hospitalization in NICU** **No** **Yes**	190 103	64.8 35.2
**Echocardiography status** Normal Abnormal	84 209	28.8 71.3
**Mother Education ** Under diploma degree Diploma degree Bachelor's degree Master's degree Doctoral degree	186 63 33 13 1	65.5 21.5 11.3 4.4 0.3
**Father Education** Under diploma degree Diploma degree Bachelor's degree Master's degree Doctoral degree	154 78 36 23 2	52.6 26.6 12.3 7.8 0.7
Mean week that Childbirth occurred	37.63±2.55	
Mean mother's hospitalization during pregnancy	0.32±0.2	
Mean mother's marriage age	21.66±5.13	
Mean father's marriage age	26.56±5.68	
**Smoking mother** **No** **Yes**	281(95.9) 12(4.1)	
**Smoking father** **No** **Yes**	201(68.6) 92(31.4)	
**Mean Baby's birth weight (g) **	2994.18±732.02	
**Mean mother's age in pregnancy **	27.78±4.88	
**Mean mother's weight before pregnancy (kg) **	68.40±12.44	
**Mean maternal weight gain during pregnancy (kg) **	9.74±4.83	
**Mean number of pregnancy (parity) **	1.99±1.16	
**Mean LVEF**	63.03±3.94	
**Mean LVFS**	33.41±3.5	

**Table:** Table2. Echocardiographic profile of congenital heart diseases

Congenital Heart Diseases	N= 209	(100%)
Patent Foramen Ovale (PFO)	51	24.4%
Atrial septal defect (ASD)	40	19.13%
Patent ductus arteriosus (PDA)	11	5.26%
Tetralogy of Fallot (TOF)	9	4.3%
Coarctation of the aorta (TOA)	3	1.43%
Aortic stenosis (AS)	2	0.95%
Truncus arteriosus (TA)	17	8.13%
Bicuspid valve aortic (BVA)	2	0.95%
Arterial Insufficiency (AI)	2	0.95%
Pulmonary Insufficiency (PI)	4	1.91%
Pulmonary Stenosis (PS)	5	2.39%
Multiple Sclerosis (MS)	2	0.95%
Ventricular septal defect (VSD)	8	3.82 %
Pulmonary hypertension (PH)	7	3.34%
Left atrial enlargement (LAE)	11	5.26%
Left Ventricular Enlargement (LVE)	7	3.34%
Left Ventricular Hypertrophy (LVH)	14	6.6%
Right Atrial Enlargement (RAE)	5	2.39%
Right Ventricular Exclusion (RVE)	5	2.39%
Hypertrophic Cardiomyopathy (HCM)	2	0.95%
Atrioventricular block (AVB)	2	0.95%

A total of 293 children were enrolled in this study. Males exhibited a significantly higher prevalence of CHD (59.72%) compared to females (40.27%). Nearly half (45%) of the participants were under one year of age. Furthermore, 64.8% of the patients had no prior admissions to the neonatal intensive care unit (NICU), while 71.3% presented with abnormal echocardiographic findings. In the study of parents' education, it was found that most of the mothers (65.5%) and fathers (52.6%) are under the diploma and most of the mothers (25.9%) and fathers (68.6%) do not smoke. The mean LVEF and LVFS for the study population were 63.03 ± 3.94 and 33.41 ± 3.5, respectively (Table-1).

Table-1 presents the demographic and clinical information of 293 patients. Analyzing each of these variables allows us to identify patterns related to different disease states and influencing factors. Among the 293 patients, 175 (59.72%) were male, and 118 (40.27%) were female. This ratio indicates an unequal gender distribution, which could be due to various factors such as biological differences, cultural influences, or even access to medical services. The majority of patients (45%) were under one year of age, showing that infants and very young children make up the largest proportion of the patient population. In other age groups, the proportion of patients is lower, for instance, 8.9% are aged 1-3 years, 11.6% are aged 3-6 years, 24.6% are aged 6-12 years, and 9.9% are aged 12-15 years. This distribution highlights the greater vulnerability of infants, possibly due to their increased susceptibility to infections and cardiovascular issues (Table-1). As for NICU hospitalization, 64.8% of patients were not admitted to the NICU, while 35.2% required admission. This statistic shows that about one-third of these children needed specialized care, potentially due to critical conditions at the time of admission. Among the patients, 209 (71.3%) had abnormal echocardiography results, while only 28.8% showed normal results. This clearly demonstrates that the majority of the patients were dealing with cardiac issues. The mean LVEF in this group was reported to be 63.03±3.94, indicating a satisfactory and normal level. Given the p-value of less than 0.001, this result is statistically significant. Additionally, the mean LVFS and LVEF were 33.41±3.5 and 63.03±3.94, respectively (Table-1).

Among 209 cases of CHD, PFO constituted 51 cases accounting for 24.4% of all CHD cases. ASD was the second most common CHD accounting for 19.13% of all CHD cases. TA constituted 17 cases accounting for 8.13% of all cases (Table-2).

According to Table-3, by using t-test analysis, there is no statistically meaningful difference between the two groups of ECH and without ECH patients in studied variable. As the Table shows, only LVFS was significant among the groups (P=0.029).

Examining maternal health characteristics, 73.3% (245 of 293) of the mothers reported no history of pre-existing chronic medical conditions. Hypothyroidism or hyperthyroidism emerged as the most prevalent maternal co-morbidity, affecting 9.9% (29 cases) of the participants. Diabetes mellitus (type 1 or 2) was present in 15.1% (15 cases). In terms of medication use during pregnancy, over two-thirds (70.3%, 206 cases) of mothers reported no prior medication use. Levothyroxine, prescribed for thyroid conditions, was the most frequently used medication during gestation, accounting for 8.5% of cases. Statistically significant associations (P<0.001) were identified between maternal factors and the presence of abnormal echocardiographic findings in their children. These factors included a history of COVID-19 infection during pregnancy, the type of delivery employed, and the child's NICU hospitalization history (P=0.015 and P=0.010, respectively).

Examining maternal health characteristics of all patients' mothers, 73.3% reported no history of pre-existing chronic medical conditions. Hypothyroidism or hyperthyroidism emerged as the most prevalent maternal co-morbidity, affecting 9.9% of the participants. Diabetes mellitus (type 1 or 2) was present in 15.1%. In terms of medication use during pregnancy, over two-thirds (70.3%) of mothers reported no prior medication use. Levothyroxine, prescribed for thyroid conditions, was the most frequently used medication during gestation, accounting for 8.5% of cases. Statistically significant associations (P<0.001) were identified between maternal factors and the presence of abnormal echocardiographic findings in their children. These factors included a history of COVID-19 infection during pregnancy (P<0.001), the type of delivery employed, and the child's NICU hospitalization history (P=0.015 and P=0.010, respectively)(Table-4).

Moreover, univariate and multivariate logistic regression models were used to investigate factors affecting ECH. First, the effect of each variable was investigated using the univariate logistic regression model, and the variables such as mother's weight before pregnancy, Mother's infection with Covid-19 during pregnancy, Mother's History medicine usage, week that Childbirth occurred, type of delivery, Hospitalization in NICU, Father Education) that had a p-value less than 0.2 were entered into the multivariate logistic regression model.

Multivariate logistic regression model with Backward method demonstrated that COVID-19 infection during pregnancy and the type of delivery, caesarian delivery, fathers' education (under diploma degree) have statistically significantly effect on echocardiographic status.

The chance of having ECH for children whose mothers were infected with Covid- 19 during pregnancy is 6.73 times more than of children whose mothers did not have covid- 19. (OR=6.73).

The chance of having ECH for children who were born with caesarian delivery is 1.77 times more than of children born with vaginal delivery (OR=1.77)

The chance of having ECH for children whose fathers' education was under diploma degree is 2.56 times more than other children (OR=2.56)

## Discussion

**Table:** Table3. Mean differences of two groups of ECH and without ECH in studied variables

Variables	Mean Difference	SE	t	P-value
**Mother's age in pregnancy**	0.26	0.89	0.29	0.77
**Number of pregnancy (parity)**	0.16	0.89	1.04	0.299
**Mother's weight before pregnancy (kg)**	-2.38	1.61	-1.47	0.142
**Maternal weight gain during pregnancy (kg)**	0.45	0.63	0.71	0.478
**Mother's hospitalization during pregnancy**	-0.14	0.9	-1.46	0.147
**Mean mother's marriage age**	0.41	0.67	0.62	0.539
**Mean father's marriage age**	0.89	0.76	1.18	0.241
**Mother's age in pregnancy**	0.28	0.98	0.28	0.778
**Week that Childbirth occurred**	0.42	0.33	1.28	0.201
**Baby's birth weight**	95.91	94.63	1.014	0.312
**LVEF**	0.39	0.51	0.77	0.444
**LVFS**	1.02	0.45	2.028	0.029*

**Table:** Table4. Distribution and comparison of studies variables in patients with normal and abnormal echocardiography

	**N=293** **(%)**	**Patients with normal ECH (n=84)**	**Patients with abnormal ECH** **(n=209)**	**P-value**
**Mother's disease history ** Without disease Heart disease Lupus Diabetes Type 1 and 2 Seizure Asthma Hypothyroidism/ hyperthyroidism Hypertension	215(73.3) 6(2.0) 2(0.68) 15(5.1) 5(1.7) 4(1.3) 29(9.9) 17(5.8)	73(86.9%) 2(2.4%) 0(0/0) 0(0.0) 0(0.0) 8(9.5%) 7(8.3%) 1(1.2%)	180(86.1%) 4(1.9%) 1(0.5%) 6(2.9%) 1(0.5%) 21(10.0%) 23(11.0%) 2(1.0)	0.942
**Mother's surgical history** No Yes	193(65.9) 100(34.1)	60(71.4%) 24(28.6%)	133(63.6%) 76(36.4%)	0.224
**Mother's infection with Covid-19 during pregnancy** No Yes	222(75.8) 71(24.2)	79(35.5%) 5(6.0%)	131(64.7%) 66(31.6%)	<0.001
**Preeclampsia** Normal Abnormal	276(94.2) 17(5.8)	79(94.0%) 5(6.0%)	197(94.3%) 12(5.7%)	0.944
**Use of complementary medicine for pregnancy** No Yes	3(1.0) 290(98.9)	1(1.2%) 83(98.8%)	1(1.2%) 210(99.0)	0.655
**Mother's History medicine usage** No Methyldopa Corton Aspirin Insulin Antibiotics Levothyroxine Anticonvulsants Metformin	206(70.3) 15(5.1) 19(6.5) 1(0.3) 9(3.1) 10(3.4) 25(8.5) 1(0.3) 6(2.0)	67(33.5%) 5(6.0%) 1(1.2%) 0(0.0) 0(0.0) 2(2.4%) 7(8.3%) 0(0.0) 2(2.4%)	139(69.5%) 10(4.8%) 18(8.6%) 1(0.5) 9(4.3%) 8(3.8%) 18(8.6%) 1(0.5%) 4(1.9%)	0.192
**Type of delivery** Vaginal delivery Caesarean section	128(43.7) 165(56.3)	45(52.8%) 38(45.3%)	82(39.2%) 127(60.8)	0.015
**Number of pregnancies** No Yes	276(94.2) 17(5.8)	80(95.2%) 4(4.8%)	196(93.8%) 13(6.2%)	0.786
**Patient's NICU Hospitalization** No Yes	190(64.8) 103(35.2)	64(76.2%) 20(23.8%)	126(60.3%) 83(39.7%)	0.010

Variations in reported congenital heart disease (CHD) prevalence likely arise from discrepancies in detecting minor anatomical defects, particularly small ventricular septal defects (VSDs), which can close spontaneously during infancy [15]. This study aimed to analyze the echocardiographic characteristics, prevalence, and associated risk factors of CHDs within the northwest region of Iran. Our investigation revealed a prevalence of specific CHD types, particularly noting that patent foramen ovale (PFO) was identified as the most common anomaly (81%), while atrial septal defect (ASD) emerged as the second most prevalent (56%). This contrasts with existing literature where VSDs are often reported as the most frequent anomaly [24][25]. Prior research suggests a potential association between sex at birth and the type and presence of CHD, indicating sex as a possible risk factor [16][17]. Our findings support these observations, demonstrating a significantly higher prevalence of CHD in male children [18][19][20]. For instance, Cao et al. reported a statistically significant increase in CHD incidence among male neonates (9.96 per 1,000 live births compared to 7.34 in females) [20], a trend mirrored in our data. Similarly, Wu et al. observed a higher global CHD rate in male neonates (19.1 vs. 16.6 per 1,000) over a two-decade period [21]. While our findings regarding sex distribution differ from some studies that suggest a higher prevalence of specific CHDs (such as VSD, patent ductus arteriosus (PDA), and ASD) in females [22][23], they highlight potential regional variations. For example, Andishmand et al. reported a higher prevalence of these specific CHDs in female infants from southern Iran [22], while Ishikawa et al. noted a slight female predominance in VSD and PDA prevalence within their Chinese patient population [23]. These discrepancies in sex distribution indicate a need for further regional studies to understand the varying patterns in CHD prevalence. The discrepancy in our findings regarding PFO as the most prevalent anomaly (81%) compared to prior studies (e.g., Mesa et al. reported a significantly lower prevalence of 12.8% for PFO as the second most common type) [26] warrants attention. Our results align with findings from Wiktor et al. and Rao et al., who also reported high prevalence rates for ASD [27][28]. It is important to note that ASDs often go undetected during childhood, which may contribute to their higher prevalence in adult populations. Existing literature suggests a range of 4.2 to 8.6 per 1,000 live births for CHD prevalence in Iranian populations [29]. In line with Rahim et al.'s investigation conducted in western and southern Iran, our study identified ASD as the most prevalent congenital heart defect among 293 participants [30]. Moreover, aligning with findings by Kishore et al., nearly half (45%) of the CHD cases in our study were diagnosed within the first year of life [31]. This higher frequency in younger age groups may be attributed to advancements in prenatal detection methods for suspected CHDs. Early diagnosis enables timely intervention and potentially mitigates negative health outcomes associated with delayed diagnosis [32]. Extensive research has established potential risk factors for CHD, including both maternal and paternal age extremes, as well as low socioeconomic status [33]. Commonly cited maternal health risk factors during pregnancy include diabetes, insulin use, systemic lupus erythematosus, hypertension, obesity, unspecified febrile illnesses, specific infections (e.g., rubella and parvovirus), smoking, and gestational stress [34]. Our investigation identified statistically significant associations between specific factors—such as maternal COVID-19 infection during pregnancy, the type of delivery employed, and a history of NICU hospitalization—and abnormal echocardiographic findings suggestive of CHD in children. These findings indicate that maternal health during pregnancy may influence the prevalence of CHDs, supporting the need for further research to elucidate the underlying mechanisms. An analysis of maternal health revealed underlying medical conditions in 26.6% of the mothers participating in this study. Hypothyroidism or hyperthyroidism emerged as the most prevalent pre-existing condition (9.9%), followed by hypertension (5.8%) and diabetes mellitus (5.1%). Established research demonstrates a significant increase in the risk of congenital cardiac anomalies in offspring born to mothers with uncontrolled diabetes [35][36], which may include hypertrophic cardiomyopathy, VSDs, or transposition of the great arteries. Conversely, optimal glycemic control prior to conception and throughout pregnancy can reduce the risk of CHD in offspring [37]. These findings underscore the potential effectiveness of educational programs designed to empower mothers regarding diabetes management, which could lead to a decrease in the prevalence of congenital heart disease in newborns. Childhood diseases hold special importance as they can have long-lasting effects on a child's health and quality of life. Early diagnosis and treatment of these conditions play a critical role in preventing serious complications. Focusing on preventive care and addressing risk factors during pregnancy and early childhood is essential to reduce the prevalence of such diseases [38][39][40][41][42].

Limitation of study

This study acknowledges several limitations. First, the focus on neonates referred for diagnosis and treatment potentially underrepresent the true prevalence of CHD in the population. This is because critically ill neonates who may have succumbed during resuscitation efforts before undergoing echocardiography were not captured in our data. Second, the absence of pulse oximetry screening results for children not referred for cardiology evaluation limits our ability to quantify the test's sensitivity, specificity, and false positive rates within this specific context. Finally, environmental risk factors were not investigated in this study. Given the lack of a standardized global system for CHD data collection, further detailed epidemiological studies are warranted to elucidate potential regional variations in both the prevalence of CHD and associated risk factors.

## Conclusion

Our investigation identified PFO as the most prevalent congenital heart defect, followed by ASD. Furthermore, a statistically significant higher prevalence of CHD was observed in male children. Nearly half (45%) of diagnosed CHD cases were detected within the first year of life. Significantly, the study established associations between specific factors and abnormal echocardiographic findings suggestive of CHD in children. These factors included maternal COVID-19 infection during pregnancy, the type of delivery employed, and a history of NICU hospitalization. These findings warrant further research to confirm causal relationships and elucidate the underlying mechanisms by which these factors may contribute to CHD development. This data can serve as a valuable resource for stakeholders in developing targeted public health policies and interventions to enhance early detection and management strategies for CHD in the pediatric population.

## Conflict of Interest

The authors have no conflicts of interest relevant to this article to disclose.

## Acknowledgment

The authors would like to express their gratitude to the clinical research development unit of Imam Khomeini Hospital, Urmia University of Medical Sciences, for English writing and editing.
